# Abdominal Pain and Ascites: Not Always Related to Portal Hypertension

**DOI:** 10.7759/cureus.812

**Published:** 2016-10-03

**Authors:** Fredy Nehme, Gilbert Kisang, Michael Green, Nathan Tofteland

**Affiliations:** 1 Department of Internal Medicine, Kansas University School of Medicine - Wichita; 2 Department of Medicine and Pediatrics, Kansas University School of Medicine - Wichita

**Keywords:** eosinophilic ascites, eosinophilic gastroenteritis, gastrointestinal disorder

## Abstract

Eosinophilic gastroenteritis is a rare inflammatory disorder of the gastrointestinal tract with an estimated prevalence of one in 100,000. The typical presentation consists of vague gastrointestinal symptoms with the mucosal involvement of the digestive system. Rarely, it presents as eosinophilic ascites. We report the case of a 22-year-old female who presented with acute onset abdominal pain and ascites. The laboratory studies were remarkable for eosinophilia and the ascitic fluid demonstrated high eosinophilic counts. Push enteroscopy with biopsy supported the diagnosis of eosinophilic gastroenteritis, with likely serosal involvement. Other differential diagnoses were excluded. A prednisone taper along with dietary treatment was initiated. We report complete resolution of symptoms two weeks following the initiation of therapy. Nine months later, she remains asymptomatic without recurrence of ascites.

## Introduction

Eosinophilic gastroenteritis (EGE) is an uncommon condition characterized by eosinophilic infiltration of various layers of the gastrointestinal (GI) tract, with an estimated prevalence of one in 100,000 [1]. To date, no diagnostic criteria have been established and the diagnosis is made by exclusion. Its presentation is vague and can mimic a variety of GI disorders such as inflammatory bowel disease, GI infections and irritable bowel syndrome [2]. Eosinophilic ascites is an unusual presentation of eosinophilic gastroenteritis, occurring when there is serosal involvement of the affected section of the bowel [1]. Atopy and food allergies are noted in 50 to 70% of the cases [2]. We report a case of eosinophilic ascites in a patient without a history of seasonal allergies, food allergies or asthma. Informed consent was obtained from the patient for this study.

## Case presentation

**Figure 1:**
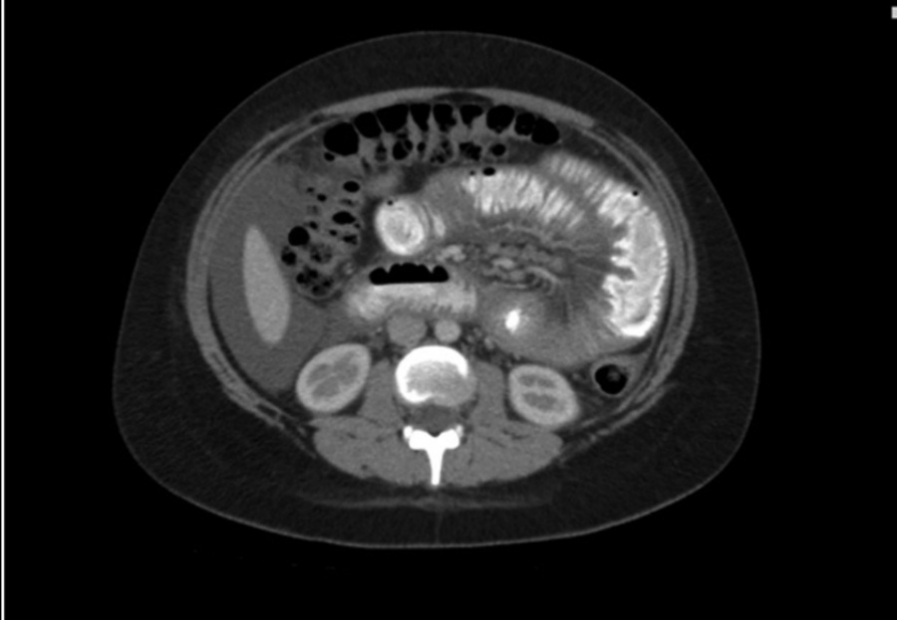
CT scan of the abdomen Diffuse bowel wall thickening with extensive abdominal and pelvic ascites.

A 22-year-old female patient with a history of depression presented to the emergency department with a one-week history of epigastric abdominal pain, abdominal distention, constipation, and bloating. She denied recent fever, chills, night sweats, weight loss, sick contacts, and recent travel. Home medication included only fluoxetine. She had no history of allergies, asthma, arterial or venous thromboembolism. A review of systems was negative for cardiac, neurologic or renal involvement. A physical examination revealed moderate abdominal distension and tenderness. The initial abdominopelvic computed tomography (CT) scan showed small intestinal mucosal thickening suggestive of enteritis with a moderate amount of free pelvic fluid. Symptomatic treatment was initiated and the patient was discharged home. Due to worsening abdominal pain and distention, she was readmitted two weeks later. The laboratory investigation was significant for leukocytosis of 18.9 k/µL with peripheral blood eosinophilia of 8.8 k/µL (47%). The liver profile and total protein counts were within normal limits. A repeat CT scan showed extensive abdominal and pelvic ascites, dilated jejunal loops and mucosal thickening, with a few slightly prominent mesenteric lymph nodes, and mild bilateral pleural effusions (Figure 1). No air-fluid levels or other signs of small bowel obstruction were noted. An ultrasound guided abdominal paracentesis demonstrated a low serum to ascites albumin gradient of 0.3 g/dL (albumin 2.8 g/dL, simultaneous serum albumin 3.1 g/dL), with significantly elevated white blood cells (WBCs) (6900/mm^3^) and > 90% eosinophils. Evaluation for underlying infectious causes, including stool cultures, Giardia, Strongyloides, Entamoeba histolytica, human immunodeficiency virus (HIV), Toxocara, and Cryptosporidium were negative. The antineutrophil cytoplasmic antibodies (ANCA) testing was negative, while immunoglobulin E (IgE) was normal at 18 IU/mL and tryptase was 11.5 ng/mL.

**Figure 2:**
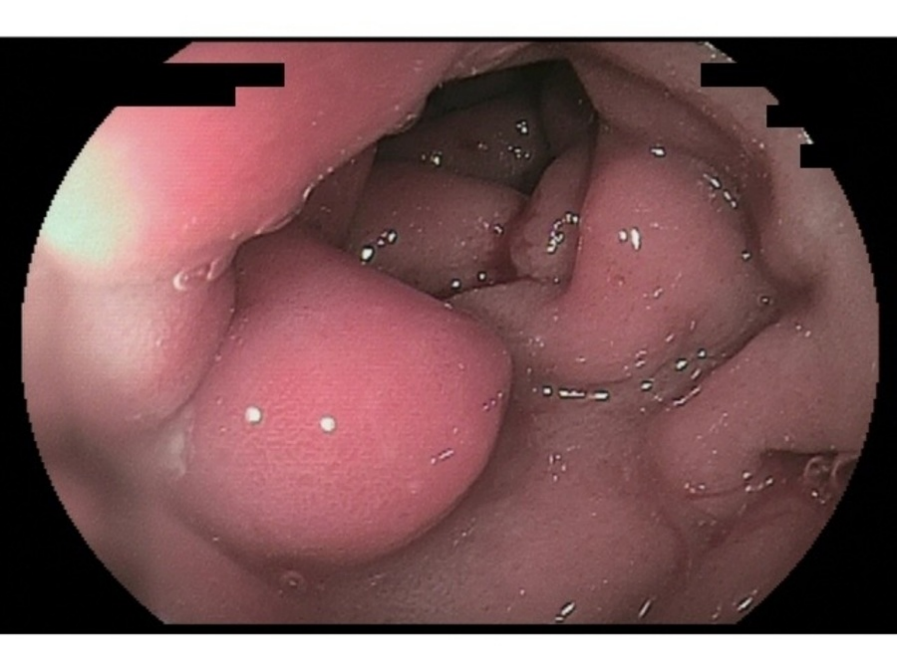
Push enteroscopy revealing diffuse erythema and edema along the gastrointestinal tract

**Figure 3:**
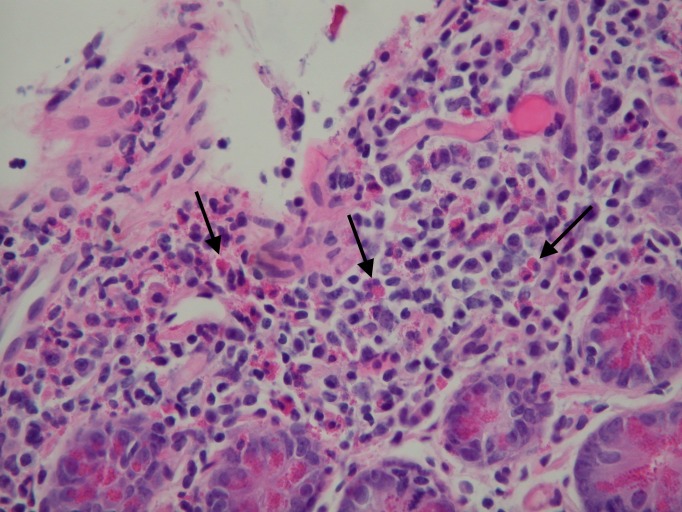
Cytology specimen showing clusters of eosinophils in the deep lamina propria and crypt epithelium Hematoxylin and eosin (H&E) stain, X400 magnification (eosinophils designated by the black arrows).

Given the radiographic suspicion of an inflammatory process involving the jejunum, she subsequently underwent push enteroscopy that revealed gastric antral erythema, edematous mucosa of the duodenal bulb, and scattered jejunal erythema and edema (Figure 2)*. *The histology showed extensive eosinophilic involvement reaching the deep lamina propria of the jejunum, duodenum, stomach and esophagus, reaching up to 74 eosinophils/high-power field, consistent with EGE (Figure 3)*. *

The diagnosis was made after other causes of eosinophilia and eosinophilic gastrointestinal infiltration was excluded. Parasitic infection was excluded based on the stool studies and serologic testing. Gastrointestinal malignancy and inflammatory bowel disease were ruled out based on endoscopy and biopsy results. Hypereosinophilic syndrome was unlikely given a lack of other organ involvement. Polyarteritis nodosa was ruled out with biopsy results negative for perivascular eosinophilia. Eosinophilic granulomatosis with polyangiitis is very unlikely given a negative ANCA and the absence of a history of asthma.

She was started on oral prednisone 40 mg tapered over six weeks and an empiric six-food elimination diet (fish, milk products, nuts, shellfish, soy products, and wheat). A two-week follow-up showed major improvement of her abdominal pain, ascites, and eosinophilia (eosinophilic count of 0.29 k/µL five days after treatment). A long-term follow-up nine months after presentation continued to show complete resolution of her symptoms and eosinophilia while the patient continued to follow the six-food elimination diet.

## Discussion

Eosinophilic gastrointestinal disorders (EGID) constitute a spectrum of disorders characterized by eosinophilic infiltration of different segments of the GI tract. Eosinophilic gastroenteritis (EGE) is part of the spectrum of EGID, with typically affected sites being the stomach and the small bowel. However, it can include any area of the GI tract from the esophagus to the rectum. The symptoms vary depending on the location and depth of the involvement [3]. EGE has three subtypes: mucosal, muscular, and subserosal depending on the clinical picture and the depth of infiltration within the GI wall [4]. The rarest presentation remains the serosal form (12% of cases), as eosinophils infiltrate all layers of the digestive wall to the serosal layer, causing eosinophilic ascites [1].

To date, no diagnostic criteria have been defined for EGE. The diagnosis is based on a high index of suspicion when patients present with GI symptoms along with laboratory and pathological findings suggestive of GI tract infiltration by eosinophils [5].

The relatively normal finding of eosinophilia within the GI tract [6], along with the rarity of the disease, have led to the absence of consensus on histological features with no predefined cut-off for the number of eosinophils/high-power field. Upper endoscopy is usually sufficient to establish a diagnosis, with multiple biopsies taken from both normal and abnormal areas [7]. If mucosal biopsies remain negative in the presence of a high index of suspicion, a laparoscopic full-thickness biopsy is suggested to establish a diagnosis [8].

A high heterogeneity of dietary management options has been reported with an overall effectiveness in inducing clinical remission or improvement in 87.2% of children and 88% of adults [9].

Corticosteroids constitute the main treatment option in patients for whom dietary treatment was not feasible. After the initial response, the drug is usually tapered over a few weeks [10].

This patient had EGE with involvement of the esophagus, stomach, duodenum, and jejunum as confirmed by histopathological examination. No personal or family history of allergies was found and the IgE level was normal on presentation. The pathology results showed clusters of eosinophils reaching to the deep margin of the mucosal biopsies. Given her presentation with eosinophilic ascites, pleural effusion, and significant eosinophilia, serosal involvement is likely. A laparoscopic full-thickness biopsy was not obtained given the invasive nature of the procedure and her prompt response to therapeutic intervention. The patient had an excellent response to steroid therapy and dietary treatment, with resolution of symptoms and eosinophilia several days after therapy. Although there are no head-to-head trials comparing available dietary treatments, most of them showed comparable response rates [9]. The dietary modality used in our case was an empiric six-food elimination diet. A recent meta-analysis estimated an 85.3% symptomatic improvement for EGE patients given this dietary treatment [9].

## Conclusions

EGE is a rare but highly curable disease, diagnosed by maintaining a high index of suspicion when presentation includes GI symptoms and peripheral eosinophilia. Eosinophilic ascites is an unusual presentation of EGE particularly associated with serosal involvement of the GI tract. This presentation is characterized by very high peripheral eosinophilia and an excellent response to steroids. This report adds to the limited data available on extraintestinal involvement in EGE.

## References

[1] Talley NJ, Shorter RG, Phillips SF, Zinsmeister AR (1990). Eosinophilic gastroenteritis: a clinicopathological study of patients with disease of the mucosa, muscle layer, and subserosal tissues. Gut.

[2] Sheikh RA, Prindiville TP, Pecha RE, Ruebner BH (2009). Unusual presentations of eosinophilic gastroenteritis: case series and review of literature. World J Gastroenterol.

[3] Zhang MM, Li YQ (2016). Eosinophilic gastroenteritis: a state-of-the-art review. J Gastroenterol Hepatol.

[4] Ingle SB, Hinge Ingle CR (2013). Eosinophilic gastroenteritis: an unusual type of gastroenteritis. World J Gastroenterol.

[5] Lopez-Medina G, Gallo M, Prado A, Vicuna-Honorato I, Castillo Diaz de Leon R (2015). Eosinophilic Gastroenteritis: case report and review in search for diagnostic key points. Case Rep Gastrointest Med.

[6] Tan AC, Kruimel JW, Naber TH (2001). Eosinophilic gastroenteritis treated with non-enteric-coated budesonide tablets. Eur J Gastroenterol Hepatol.

[7] Lee M, Hodges WG, Huggins TL, Lee EL (1996). Eosinophilic gastroenteritis. South Med J.

[8] Hoe LV, Vanghillewe K, Baert AL, Ponette E, Geboes K, Stevens E (1994). CT findings in nonmucosal eosinophilic gastroenteritis. J Comput Assist Tomogr.

[9] Lucendo AJ, Serrano-Montalban B, Arias A, Redondo O, Tenias JM (2015). Efficacy of dietary treatment for inducing disease remission in eosinophilic gastroenteritis. J Pediatr Gastroenterol Nutr.

[10] Zhang L, Duan L, Ding S, Lu J, Jin Z, Cui R, McNutt M, Wang A (2011). Eosinophilic gastroenteritis: clinical manifestations and morphological characteristics, a retrospective study of 42 patients. Scand J Gastroenterol.

